# The Short Tandem Repeat of the *DMT1* Gene as a Molecular Marker of Elite Long-Distance Runners

**DOI:** 10.1155/2019/7064703

**Published:** 2019-11-23

**Authors:** Gerile Wuyun, Yang Hu, Zihong He, Yanchun Li, Xu Yan

**Affiliations:** ^1^Institute of Sport, Inner Mongolia Normal University, China; ^2^China Institute of Sport and Health Science, Beijing Sport University, China; ^3^Biology Center, China Institute of Sport Science, China; ^4^Institute for Health and Sport, Victoria University, Australia; ^5^Australian Institute for Musculoskeletal Sciences, Australia

## Abstract

The *DMT1* gene encodes divalent metal transporter 1, a membrane iron transport protein. Divalent metal transporter 1 influences cellular iron availability, which might further affect aerobic exercise capacity. Short tandem repeat (STR) polymorphisms have been used as genetic markers in the literature, yet the STR polymorphisms of the *DMT1* gene have not been well studied. In this current study, we explored the polymorphisms of the *DMT1* gene in a group of elite long-distance runners and controls, by using the PCR-RFLP (Restriction Fragment Length Polymorphism) and Gene scan technology. We found that the genotype frequency of the homozygous 258 bp STR polymorphism of the *DMT1* gene (258 bp/258 bp) was significantly higher in the athlete group than in the controls (*χ*^2^ = 14.01, *p* = 0.006) so does the allele frequency of the 258 bp STR polymorphism (*χ*^2^ = 12.867, *p* = 0.008). These data suggested that the STR polymorphism of the *DMT1* gene might be correlated with aerobic exercise capacity and the 258 bp homozygous (25 bp/258 bp) could be used as a molecular marker for the talent identification of elite long-distance runners.

## 1. Introduction

Divalent metal transporter 1 (DMT1) is a major membrane transport protein, which uptakes nonheme iron within the lumen [1]. The *DMT1* gene is expressed in many human tissues, such as the small intestine, kidney, liver, and lung [2]. It is known that the human proximal small intestine is the main site for iron absorption, in which iron element exists in the form of Fe^2+^. Fe^2+^ could enter into blood circulation via the mediation of Ferroportin1 (FPN1) and Hephaestin (HP) or enter into epithelial cells with the mediation of DMT1 [3]. Iron element plays an important role in constituting Hemoglobin (Hb) and Myoglobin (Mb). Therefore, iron element is important for exercise, yet its function can be characterized by duality [4]. A deficiency of iron element affects the maximal uptake of oxygen (VO_2_max) of athletes and is associated with a decreased exercise capacity, due to the reduction in protein synthesis, the decreased oxygen transporting and storing capacity, and the fall of enzymatic activities and hormones level, as well as the reduction in phosphorylation capacity and decreased adenosine triphosphate (ATP) production [5]. On the other hand, excessive iron element also affects exercise ability. Free iron element induces the production of free radicals, leading to an increase of human lipid peroxidation level and even tissue damage. Thus, both iron deficiency and iron excess can directly affect exercise ability [6].

Human exercise ability has distinct individual differences, which have been attributed from both the Nurture side (diet and training) [7, 8] and the Nature side (genetics) [9–16]. The human *DMT1* gene is located in 12q13, with 17 exons. The expression of the *DMT1* gene depends on the basis of the organism or cell iron status [17]. Lee et al. first discovered 5 polymorphic sites in the third intron of the human *DMT1* gene, as well as 1 short tandem repeat (STR) microsatellite polymorphism [18]. STR is a short tandem repeat sequence, with its core sequence 2-6 bp in length. STR mainly exists in the noncoding regions of the genome, which is related to the genetic recombination and gene express regulation, and maintains genome stability [19]. The STR located in the gene exon region has been reported to encode protein [20]. STR is widely existed in the genome, with a low recombination rate. Because of its compliance with Mendelian Inheritance, i.e., its codominant heredity, STR is widely used in association studies including genetic phenotype, gene mapping, genetic disease diagnosis, and paternity tests. It is therefore known as the second generation of genetic markers.

In this current study, we explored the STR microsatellite polymorphisms of the *DMT1* gene in elite long-distance runners and the controls, with the aim to identity molecular genetic markers for talent identification of elite long-distance runners.

## 2. Materials and Methods

### 2.1. Research Subjects

The study has been approved by the ethics committee of Beijing Sport University; all the participants have signed a consent form. The study has been conducted according to the principles of the Declaration of Helsinki [21].

For the athlete group, there are 123 elite long-distance runners, all from the northern region of China, all in good health, and all belonging to Han nationality. The details are shown in Table 1.

For the control group, there are 102 college students from Beijing Sport University, without any professional training, all from the northern region of China, all in good health, and all belonging to Han nationality. The details are shown in Table 2.

### 2.2. Experimental Method

**Figure 1:**
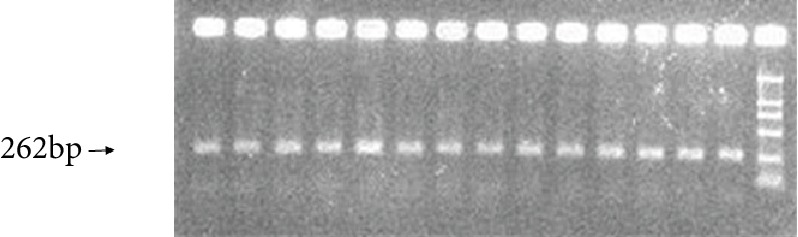
A presentative agarose picture of the PCR product of the *DMT1* gene STR polymorphism.

DNA was extracted using the Wizard® Genomic DNA Purification Kit (A1125, Promega, Wisconsin, USA). The STR polymorphism (rs2076732) of the *DMT1* gene was obtained from the NCBI SNP library. The STR polymorphism is located in intron 3 of the *DMT1* gene, with the repetitive sequence: (TA) 6/7 (CA) 11/12/13 CCCCATCTA (TATC) 3 (TCTG) 4/5 TCCG TCTA 5/6/7/9/10. The PCR primers were designed with the Primer 5.0 software and were synthesized by Sangon Biotech (Shanghai, China). The primer sequences are as follows: forward primer: 5′-ggaTGGCTCaagTTCagcag-3′ (TAMRA fluorescent tags 5′ item); reverse primer: 5′-TGGGCTatgGTTGTGccaCTG-3′. The PCR amplification fragments are 262 bp in length (Figure 1).

**Figure 2:**
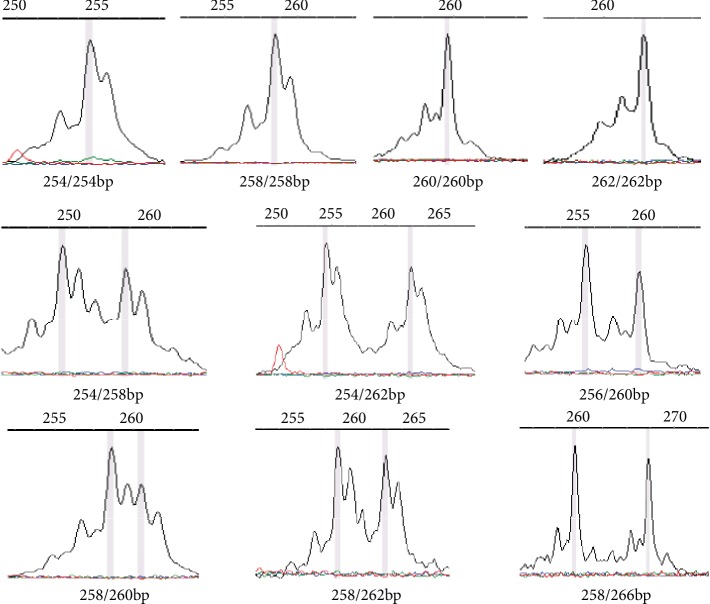
Representative picture of the GeneScan genotype data of the *DMT1* gene STR polymorphism.

The distribution of the *DMT1* gene STR polymorphisms was determined by the ABI 377 DNA Sequencer (Applied Biosystems, Massachusetts, USA) with the GeneScan detection technology (Figure 2).

### 2.3. Data Analysis

Data was analyzed with the SPSS15.0. A *χ*^2^ test was used to check if the genotype frequency and allele frequency are in line with the Hardy-Weinberg equilibrium and to analyze the differences of the allele frequencies and genotype frequencies between the athlete group and the control group. A *p* < 0.05 is considered significant.

## 3. Results

A Hardy-Weinberg equilibrium was obtained for each genotype distribution frequency (*p* > 0.05, data were shown in Tables 3 and 4).

According to the *χ*^2^ test results, there were significant differences in the 258 bp/258 bp genotype frequency between the athlete group and the control group (*χ*^2^ = 14.011, *p* = 0.006). When the athletes were further categorized as the 5, 10 km group or the marathon group, the 258 bp/258 bp genotype frequency was significantly different between the 5, 10 km group and the control group (*χ*^2^ = 11.910, *p* = 0.017) but did not reach significance between the marathon group and the control group (*χ*^2^ = 5.762, *p* = 0.217). We then checked whether there was a gender difference in the genotype frequency of the *DMT1* STR polymorphism; we did not find a significant difference between males and females among the athlete group or the control group. However, we did found that the 258 bp/258 bp genotype frequency was significantly higher in the male athletes than in the male controls and significantly higher in the female athletes than in the female controls.

We then checked the allele frequency of the 258 bp allele, which was significant differently between the athlete group and the control group (*χ*^2^ = 12.867, *p* = 0.008, Table 5), as well as the 5, 10 km group and the control group (*χ*^2^ = 11.082, *p* = 0.021). There was no significant difference in the frequency of the 258 bp allele between the marathon group and the control group (*χ*^2^ = 5.516, *p* = 0.212). When we further divided the athletes into males and females, the allele frequency was significantly higher in the male athletes and female athletes, when compared with the male controls and female controls, respectively.

## 4. Discussion

In this current study, we explored the STR polymorphisms of the *DMT1* gene; we have identified six alleles: 254 bp, 256p, 258 bp, 260 bp, 262 bp, and 266 bp. There are five alleles and a total of eight genotypes in the athlete group, while the control group has six alleles and ten genotypes; both groups are in line with the Hardy-Weinberg equilibrium. A previous study reported three alleles (260 bp, 262 bp, and 264 bp) in eight healthy Caucasians, which included two 260 bp homozygous, two 264 bp homozygous, three 260/262 bp heterozygous, and one 260/264 bp heterozygous [22]. This discrepancy could be due to the small sample size of the previous study (only eight individuals). It is also possible that there could be differences with the distribution of *DMT1* STR polymorphisms between different ethnic groups (Caucasians for the previous study and Asians for the current study).

Data from this current study suggested that there was a strong association between the *DMT1* STR polymorphism and likelihood of being elite long-distance runners. The frequency of 258 bp homozygous was significantly higher in the athlete group (55.29%) than in the control group (41.04%), similar for the 5, 10 km athlete group (58.70%). The expression of the *DMT1* gene has been associated with iron levels in the body, which might further affect aerobic exercise capacity. Previous studies have reported an increased iron loss in long-distance runners [23, 24]; iron loss can induce the expression of the *DMT1* gene in theduodenum cells [25], while moderate exercise can also increase the expression of the *DMT1* gene in duodenum cells [26]. Therefore, both iron deficiency and exercise can increase the expression of the *DMT1* gene, leading to an increased duodenal iron absorption and iron endosome transportation, which ensures the iron demand of the human body. In addition, the G>C transversion of the last nucleotide of the human *DMT1* gene exon has been associated with iron deficiency erythropoiesis and a decreased Hb level [27]. Fleming et al. have also found that the glycine-arginine mutation (G185R) of the *DMT1* gene reduced intestinal iron absorption in Belgrade rats [28].

No research to date has reported the function of the *DMT1* gene STR polymorphism; we reported the association of the *DMT1* gene STR polymorphism with the likelihood of being elite long-distance runners. We speculate the mechanisms of the *DMT1* gene STR polymorphism influencing aerobic exercise ability through the regulation of *DMT1* gene expression, which further influence intestinal iron absorption and iron status. It is known that iron status affects exercise performance [29]. The prevalence of iron deficiency anemia is higher in athletic populations than sedentary controls, especially in long-distance runners [29]. While a moderate iron supplementation is effective in preventing decline in the iron status of female collegiate swimmers during a competitive season [30], the STR polymorphisms might regulate *DMT1* gene expression via two possible mechanisms. (1) The STR polymorphisms might lead to alternative splicing of the *DMT1* gene. A recent study has proposed that there is a link between short tandem repeats and translation initiation site selection in humans [31]. Previous studies have reported the existence of isoforms of DMT1 mRNA [32, 33] and protein [33], suggesting alternative splicing of the *DMT1* gene. It is plausible that the STR polymorphisms lead to alternative splicing of the *DMT1* gene, which further affects the expression of the DMT1 gene. (2) *DMT1* might interact with hepcidin to control iron homeostasis. Hepcidin is the main iron regulatory hormone responsible for the maintenance of iron homeostasis, which controls the absorption of dietary iron and the distribution of iron among tissues and organs in the body [34]. Hepcidin level was reported to increase significantly 3, 6, and 24 h post exercise and then declined after that, returning to baseline at 72 h post exercise [35, 36]. Hepcidin was reported to decrease after 3 weeks of training in female runners [37] but increased after an intensified training period in well-trained female long-distance runners [38]. On the other hand, hepcidin treatment has been shown to decrease *DMT1* expression and iron uptake in intestinal cells [39] and in mouse intestines [40], suggesting the regulation of *DMT1* by hepcidin. It is possible that the STR polymorphisms affect the regulation capacity of hepcidin on *DMT1*, which leads to varied *DMT1* expression and different exercise capacity.

There are several limitations for the current study. (1) We only checked the STR polymorphisms of the *DMT1* gene, which are located at the intron region of the *DMT1* gene. There are a number of other *DMT1* polymorphisms reported previously, but we have not checked whether there is an addictive effect of different polymorphisms. There were no associations between *DMT1* 1303 C>A polymorphism in the exon region and iron overload in Chinese Parkinson's disease patients [41]. The 1254T>C polymorphism in the exon region of the *DMT1* gene has been associated with Parkinson's disease [42]. However, there is no study that checked the effects of those two polymorphisms on the expression of the *DMT1* gene. On the other hand, another intronic *DMT1* polymorphism, *DMT1* IVS4+44C>A, has been associated with increased DMT1 gene expression and iron level. The C allele of the *DMT1* IVS4+44C>A polymorphism is more prevalent in Parkinson's disease patients, a population known to have elevated iron accumulation in the substantia nigra, together with the increased expression of DMT1 gene expression [41]. The *DMT1* IVS4 C(+) allele occurred more frequently in Wilson's disease population than in the healthy controls, while iron dyshomeostasis has been characterized in Wilson's disease [43]. The CC genotype of the *DMT1* gene IVS4+44C>A polymorphism is associated with increased risk of age-related macular degeneration, a condition related to increased reactive oxygen species, which might be caused by the increased iron ions [44]. The AA genotype of the *DMT1* IVS4+44C>A polymorphism has reported a four-fold increase of the risk of iron deficiency anemia in children with celiac disease [45]. However, a previous study did not find significant differences in the prevalence of the DMT1 IVS4+44C>A polymorphism in the hereditary hemochromatosis cohort when compared with the control group [46]. It would be important to investigate the effect of more than one polymorphism of the *DMT1* gene on exercise capacity simultaneously, especially checking the possible haplotype on exercise capacity in future studies. (2) We have not checked the serum iron level or tissue iron level. There is currently no report about the effects of *DMT1* STR polymorphisms on serum iron levels, while the effects of the *DMT1* gene on serum iron level are not conclusive. Mice lacking intestinal *DMT1* gene have shown to have extremely low blood iron level [47]. However, patients with *DMT1* mutations (GTG deletion in exon 5 and G → T substitution in exon 8) have normal serum iron level but with a severe liver iron overload [48]. Another mutation of the *DMT1* gene (SCL11A2) has been associated with a slight increase of serum iron level, again with severe liver iron overload [49]. Together, these studies suggested that a change in *DMT1* gene expression could lead to dysregulation of iron level in blood and tissue, but the exact effects depend on the nature of the polymorphism or mutation. Further studies are warranted to investigate the effects of different *DMT1* polymorphisms on serum and tissue iron levels.

## 5. Conclusion

The frequencies of the 258/258 bp homozygous genotype and 258 bp alleles are significantly higher in the elite long-distance runners than in the control group; the *DMT1* gene STR polymorphism 258/258 bp genotype may be used as a molecular marker for the talent identification of the elite long-distance runners in Asian population.

## Figures and Tables

**Table 1:** Basic information of the athlete group.

Groups	No. of people	Age (year)	Height (cm)	Weight (kg)	Type of sports		Athletic level	
					5, 10 km	Marathon	International	National
Athlete (total)	123	23.10 ± 5.10	169.29 ± 7.50	55.53 ± 7.45	92	31	43	79
Athlete (male)	62	24.27 ± 11.87	174.95 ± 5.13	61.48 ± 4.77	47	15	11	51
Athlete (female)	61	20.84 ± 5.31	163.53 ± 4.56	49.48 ± 3.97	45	16	32	29

Note: data are expressed in average ± standard deviation.

**Table 2:** Basic information of the control group.

Groups	No. of people	Age (year)	Height (cm)	Weight (kg)
Controls (total)	102	20.31 ± 1.20	168.88 ± 8.11	62.84 ± 11.87
Controls (male)	52	20.29 ± 1.15	174.60 ± 5.88	68.36 ± 12.24
Controls (female)	50	20.33 ± 1.27	162.57 ± 4.86	56.80 ± 7.90

Note: data are expressed in average ± standard deviation.

**Table 3:** The genotype distribution of the *DMT1* gene STR polymorphism in the athlete group.

Groups	No. of people	Genotype (bp)								Hardy-Weinberg balance inspection		
		258/258	254/258	254/254	258/262	258/260	254/260	254/262	256/258	*χ* ^2^	*df*	*p*
Athlete (total)	123	68 (55.29)^∗^	33 (26.83)	9 (7.32)	6 (4.88)	3 (2.44)	1 (0.81)	2 (1.63)	1 (0.81)	2.00	7	0.96
Athlete (male)	62	33 (53.23)^#^	18 (29.03)	5 (8.07)	4 (6.45)	1 (1.61)	0 (0.00)	1 (1.61)	0 (0.00)	2.00	5	0.85
Athlete (female)	61	35 (57.38)^&^	15 (24.59)	4 (6.56)	2 (3.28)	2 (3.28)	1 (1.64)	1 (1.64)	1 (1.64)	2.00	7	0.96
5.10 km group	92	54 (58.70)^∗^	22 (23.91)	6 (6.52)	5 (5.43)	2 (2.17)	1 (1.09)	1 (1.09)	1 (1.09)	2.00	7	0.96
Marathon group	31	14 (45.16)	11 (35.48)	3 (9.68)	1 (3.23)	1 (3.23)	0 (0.00)	1 (3.23)	0 (0.00)	0.00	6	1.00

Note: data are shown in *N* (%); ^∗^*p* < 0.05when compared with the control group; ^#^*p* < 0.05 when compared with the males of the control group; ^&^*p* < 0.05 when compared with the males of the control group.

**Table 4:** The genotype distribution of the *DMT1* gene STR polymorphism in the control group.

Groups	No. of people	Genotype (bp)									Hardy-Weinberg balance inspection		
		258/258	254/258	254/254	258/262	258/260	254/260	254/262	256/258	262/262 258/266	*χ* ^2^	*df*	*P*
Controls (total)	134	55 (41.04)	31 (23.13)	9 (6.72)	19 (14.18)	10 (7.46)	2 (1.49)	5 (3.73)	1 (0.75)	2 (1.49)	0.00	8	1.00
Controls (male)	70	29 (41.43)	17 (24.29)	3 (4.29)	9 (12.86)	8 (11.43)	0 (0.00)	3 (4.29)	0 (0.00)	1 (1.43)	0.00	8	1.00
Controls (female)	64	26 (40.63)	14 (21.88)	6 (9.38)	10 (15.63)	2 (3.13)	2 (3.13)	2 (3.13)	1 (1.56)	1 (1.56)	0.00	9	1.00

Note: data are shown in *N* (%).

**Table 5:** The allele distribution of the *DMT1* gene STR polymorphism.

Groups	No. of people	Allele (bp)					
		254	256	258	260	262	266
Athlete (total)	123	54 (21.95)	1 (0.41)	179 (72.76)^∗∗^	4 (1.63)	8 (3.25)	—
Athlete (male)	62	29 (23.39)	0 (0.00)	89 (71.77)^#^	1 (0.81)	5 (4.03)	—
Athlete (female)	61	25 (20.49)	1 (0.82)	90 (73.77)^&^	3 (2.46)	3 (2.46)	—
5, 10 km group	92	36 (19.57)	1 (0.54)	138 (75.00)^∗^	3 (1.63)	6 (3.26)	—
Marathon group	31	18 (29.03)	0 (0.00)	41 (66.13)	1 (1.61)	2 (3.23)	—
Control (total)	134	56 (20.90)	1 (0.37)	172 (64.18)	12 (4.48)	26 (9.70)	1 (0.37)
Control (male)	70	26 (18.57)	0 (0.00)	92 (65.71)	8 (5.71)	14 (10.00)	0 (0.00)
Control (female)	64	30 (23.44)	1 (0.78)	80 (62.50)	4 (3.13)	12 (9.38)	1 (0.78)

Note: data are shown in *N* (%); ^∗^*p* < 0.05 when compared with the control group; ^∗∗^*p* < 0.01 when compared with the control group; ^#^*p* < 0.05 when compared with the male controls.

## Data Availability

The data used to support the findings of this study are available from the corresponding author upon request.
